# Sir Robert Edwards (1925–2013): Creator of the Greatest Happiness

**DOI:** 10.1371/journal.pbio.1001582

**Published:** 2013-05-28

**Authors:** Robert Winston

**Affiliations:** Imperial College London, London, United Kingdom

“Give me children; else I am as dead”. The desperate cry of Rachel to her husband Jacob, whose life is meaningless whilst she remains sterile, rings down the ages [1], a cry still heard in both developed countries and less affluent ones. Until recently, this pain has been largely ignored or belittled, as it was by Jacob.

**Figure:**
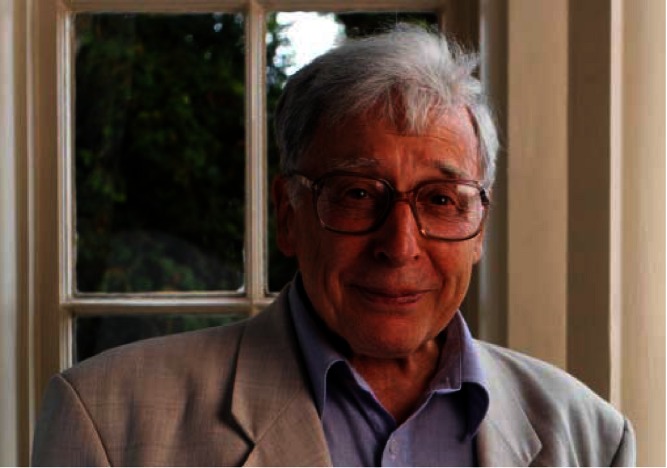
Sir Robert Edwards. Image credit: Bourn Hall Clinic.

Sir Robert Edwards, who died last week, understood it. The plight of infertile couples gave his work crucial meaning. In vitro fertilization (IVF) was not only one of the greatest medical advances of the 20th century, but it also had a profound effect on attitudes towards infertility.

Before the birth of Louise Brown, the first “test-tube” baby in 1978, infertility caused great shame. Childless couples would not talk of their plight, often not admitting it even to family. It was a deeply corrosive “stigma” with a powerful effect on every aspect of their lives.

IVF changed that. Robert Edwards, together with gynaecologist Patrick Steptoe, who died 25 years ago, pioneered a revolutionary treatment that resulted in the birth of millions of healthy children. And the immense publicity given to this truly iconic development meant that many sufferers came out of “hiding”. Ordinary people began to understand the devastating effect of involuntary childlessness.

Professor Sir Robert Edwards died April 10, 2013, at age 87. He is survived by his wife Ruth, whom he married in 1956, and by five daughters and twelve grandchildren. He was a Yorkshireman, one of three brothers, born in Batley, United Kingdom. His father, Samuel, worked on the railways and his mother, Margaret, in the local mill. Eventually the family moved to Manchester, where Edwards gained a scholarship to Manchester Central High School. Edwards never forgot his working class origins or denied his roots. He campaigned for the UK's Labour Party and was elected as a local councillor long after he had achieved his extraordinary success as a pioneering scientist.

After conscription in 1943, he entered University College of North Wales in Bangor, where he studied agriculture and zoology. In 1951, he joined the Institute of Animal Genetics in Edinburgh, where his research began under the guidance of Professor C.H. Waddington, mostly concerned with sperm function. His doctorate, a study of developing mouse embryos, was an important step. After a spell at Caltech in Pasadena, he joined the staff at Mill Hill in London, pursuing his interest in the control of ovulation. He had published work on the induction of ovulation some years earlier, and in 1963 published a study with Everett Wilson [2]. This study reported that the treatment of mice with pregnant mares' serum and human chorionic gonadotrophin (HCG) resulted in larger litters with smaller pups, many of which died. Years later, the induction of ovulation became a critical procedure for IVF, and problems concerning multiple birth and prematurity still remain. Edwards soon moved to the University of Glasgow where he studied in vitro culture of rabbit eggs, and then to Cambridge. There, funded by the Ford Foundation and working with Chris Polge, he studied the maturation of eggs from various mammalian species in vitro [3]. It isn't entirely clear from this paper if he thought about human IVF treatment, but he emphasized that these techniques would become useful in studying human fertilization and pre-implantation development.

His research flourished; often it was prescient. In 1966, he forecast the exciting possibilities offered by stem cells [4]. Much of his research foresaw the work of other researchers often employing fewer skills. In 1967, a remarkable experiment with Richard Gardner showed it was possible to sex live rabbit embryos before their transfer to the uterus [5]. He clearly saw the agricultural and human possibilities, predating by over 20 years lesser work by Richard Penketh and myself at The Royal Postgraduate Medical School. He even commented that this technique might be used in time to control sex-linked human disorders.

He began exploring the practicalities of human IVF, having described early attempts to fertilize human eggs [6]. Needing human ovaries from which he might aspirate eggs, he commuted from Cambridge to Hammersmith Hospital, then a leading gynaecological research institution. There, regrettably, he spent many fruitless hours waiting in the surgeons' room in Lower Theatre, hoping for ovarian tissue or at least an egg-containing follicle. He mostly returned to Cambridge empty-handed. I joined the Hammersmith as a junior a year later and was horrified by how my senior colleagues viewed his work with disdain. The consultant who had kept him waiting outside the theatre called it “futility” rather than fertility treatment.

Edwards heard about Patrick Steptoe and his ground-breaking work with the laparoscope. Steptoe was a gifted Oxford graduate, where he had been a music scholar as well as medical student. Although trained in leading institutions in London, he missed a plum job in a teaching hospital, and was eventually appointed as a consultant in a district general hospital, in Oldham. Steptoe was a supremely focused innovator. But in my experience, he was also sometimes aggressive in manner, and perhaps his reluctance to comply set him against the powerful medical establishment. At scientific meetings, his outstanding work with laparoscopy was often greeted with scorn. It was a long time before laparoscopy—which has revolutionized the whole of surgery—was finally accepted. Perhaps this made him an ideal partner for Edwards, particularly as the laparoscope gave them unique access to the ovary.

By 1970, together with Jean Purdy, the pair had undertaken attempts at human IVF [7]. Collaborating with others in Cambridge, notably Drs Bavister and Whittingham, and Professor C.R. Austin, Edwards tried various culture media to grow embryos, having first collected eggs after ovarian stimulation. Laparoscopic aspiration of eggs was timed after an injection of HCG, following which Edwards would make the tedious journey south, back to Cambridge.

Within a year, Edwards had grown cultured human blastocysts. These embryos seemed to have a normal complement of chromosomes and he was optimistic that the uterus would be receptive for implantation. In a paper in *Nature* in 1971, he states: “There should be no criticism in giving these [infertile] couples their own children: comments about overpopulation seem to be highly unjust to such an underprivileged minority” [8].

But controversy there was, and it grew. Not only was the clinical community largely dismissive about the possibility of successful IVF, many scientists were too. I remember a major reproduction congress in Argentina in 1974. As I, then a junior research fellow, was leaving an auditorium where Edwards had shown numerous photographs of cultured human embryos grown, I passed an eminent Cambridge professor who asked me what I thought of these pictures. I was too shy to comment. “Embryos!” he said, “[They're] not embryos—they're all dead.”

There was also, unsurprisingly, adverse comment from religious bodies. Yet years before the really virulent criticisms had started, Edwards had already delineated the key ethical issues [9]. He saw that there might be confusion in the public mind between his objectives and those supporting abortion.

Steptoe began treating severe cases of infertility, mostly women with serious pelvic damage, often suffering blocked fallopian tubes. Steptoe, who was never persuaded that tubal surgery was helpful, would offer them IVF, a new treatment that bypassed the cause of the problem. The first pregnancy was in 1976; sadly, it was ectopic [10], not uncommon in patients with tubal disease. When the pregnancy was announced, there was no shortage of criticism; some skeptical colleagues even opined that the patient was probably already pregnant at the time of embryo transfer.

Once Louise Brown was born, there was rapid and immense global interest in IVF. Medical specialists, too, recognized that this was one of the most significant developments in the history of reproduction, even though many clinicians wondered how practicable it would be to offer IVF except in a very limited manner. And at first there was no realization that Edwards had initiated what would be a huge advance for the common problem of male infertility, which previously had been largely untreatable. Lacking public research funding, Steptoe and Edwards founded the private IVF clinic at Bourne Hall near Cambridge in 1980.

Their early work at Bourne Hall faced ongoing social and religious challenges. In particular, the Conservative government of the day, under the leadership of Margaret Thatcher, failed to implement recommendations for regulating IVF that were proposed by an advisory committee headed by Mary Warnock [11], leading to concern, particularly amongst conservative members of parliament (MPs). One British MP, Enoch Powell, called embryo research “repugnant”, and introduced his Protection of the (Unborn) Child Bill in 1984, which sought to prohibit such research. Scientists and clinicians were mustered to defeat proposals such as this that might have ended IVF in the UK, but Edwards was kept somewhat in the background. An outspoken man, he was thought by some to be a bit of a loose cannon. It was Edwards's view, for example, that the appearance of the primitive streak at 14 days after fertilization was a mere arbitrary limit, challenging the advice of the Warnock Report that research should be allowed on human embryos only up to this stage; Edwards expressed doubts about banning experiments beyond this stage. This view was thought to be unhelpful, most colleagues seeing the need for such boundaries if there was to be permissive legislation.

Like all truly great scientists, bubbling with so many fresh ideas, Edwards was not always right. For a while, for example, he promoted the concept of the “helper effect”. Feeling that embryos produced a message that controlled their own implantation, he proposed that two embryos transferred to the uterus together might give a greater chance of each embryo implanting than a single embryo alone. Clinical evidence for this is still doubtful, and the irony is that multiple embryo transfer is now seen as rather risky. One of the most important complications associated with IVF is multiple births, and now there is increasing pressure to avoid it.

Latterly, Edwards was a founder of the European Society of Human Reproduction and Embryology and editor of its journal. He later established *Reproductive Medicine Online*. In this journal, Edwards sometimes seemed to deliberately provoke controversial debate—for example, giving what some thought undue prominence to one doctor who advocated human cloning. His own publication record was astonishing, with well over 350 peer-reviewed papers—of which more than 25 were in the highly prestigious journal *Nature*.

Full recognition of Edwards's work was regrettably slow. He was awarded the Lasker Prize in 2001, elected Fellow of the Royal Society in 1984, and appointed CBE, an honorary award, in 1988. It wasn't until 2010 that he was awarded the Nobel Prize for Physiology and Medicine, and the following year received a knighthood. It is shocking that the Nobel took so long. But in the 1970s, when Louise Brown was born, the World Health Organization was predicting a massive increase in world population. Wild estimates predicted that it might be greater than 110 billion; contraceptive research was seen to be more important than the treatment of infertility. And the strident voices of those who condemned work with human embryos had an effect. Such comments still rumble. Just very recently, the Society for the Protection of Unborn Children published disgraceful slurs, showing opposition still exists in a few quarters to a unique scientific advance. Robert Edwards's technology was responsible for the creation of the greatest happiness—5 million healthy lives so far and the protection of the ultimate family values. Such miserable critics might do well to consider their ethics and the biblical cry of Rachel, and reflect.
